# Atomic Adsorption Energies Prediction on Bimetallic Transition Metal Surfaces Using an Interpretable Machine Learning‐Accelerated Density Functional Theory Approach

**DOI:** 10.1002/open.202400124

**Published:** 2025-01-29

**Authors:** Jan Goran T. Tomacruz, Michael T. Castro, Miguel Francisco M. Remolona, Allan Abraham B. Padama, Joey D. Ocon

**Affiliations:** ^1^ Laboratory of Electrochemical Engineering Department of Chemical Engineering University of the Philippines Diliman Quezon City, Metro Manila 1101 Philippines; ^2^ Chemical Engineering Intelligence Learning Laboratory Department of Chemical Engineering University of the Philippines Diliman Quezon City, Metro Manila 1101 Philippines; ^3^ Institute of Physics College of Arts and Sciences University of the Philippines Los Baños Los Baños, Laguna 4031 Philippines

**Keywords:** Cheminformatics, Density functional calculations, High-throughput screening, Interpretable machine learning, Transition metal alloys

## Abstract

In this study, we identified features with the largest contributions and property trends in predicting the adsorption energies of carbon, hydrogen, and oxygen adsorbates on transition metal (TM) surfaces by performing Density Functional Theory (DFT)‐based calculations and Machine Learning (ML) regression models. From 26 monometallic and 400 bimetallic fcc(111) TM surfaces obtained from Catalysis‐hub.org, three datasets consisting of fourteen elemental, electronic, and structural properties were generated using DFT calculations, site calculations, and online databases. The number of features was reduced using feature selection and then finely‐tuned random forest regression (RFR), gaussian process regression (GPR), and artificial neural network (ANN) algorithms were implemented for adsorption energy prediction. Finally, model‐agnostic interpretation methods such as permutation feature importance (PFI) and shapely additive explanations (SHAP) provided rankings of feature contributions and directional trends. For all datasets, RFR and GPR demonstrated the highest prediction accuracies. In addition, interpretation methods demonstrated that the largest contributing features and directional trends in the regression models were consistent with structure‐property‐performance relationships of TMs like the d‐band model, the Friedel model, and higher‐fold adsorption sites. Overall, this interpretable ML–DFT approach can be applied to TMs and their derivatives for atomic adsorption energy prediction and model explainability.

## Graphical abstract

**Using Interpretative Machine Learning to Assist Density Functional Theory**: Atomic adsorption energies on 26 monometallic and 400 bimetallic transition metal surfaces were predicted by performing Density Functional Theory (DFT)‐based calculations and Machine Learning (ML) regression models. Elemental, structural, and electronic property data were obtained in online databases and in‐house calculations (see picture). The trends in these models were explained using interpretation methods, which are consistent with electron model theory.

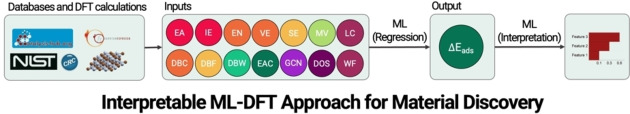

## Introduction

Energy storage and conversion technologies, including fuel cells,^[1]^ batteries,^[2]^ and carbon conversion and storage^[3]^ are developing as complementary solutions alongside the implementation of renewable energy infrastructure and facilitating the technological shift away from fossil fuels. These technologies rely on electrochemical reactions such as oxygen reduction reaction (ORR),^[4]^ oxygen evolution reaction (OER),^[5]^ hydrogen oxidation reaction (HOR),^[6]^ hydrogen evolution reaction (HER),^[6]^ and carbon dioxide reduction (CO_2_RR).^[7]^ Since the transition state energies of these reactions are linearly correlated with adsorption energies, defined as the energies needed to break the attachment between a reactant adsorbate and a surface,^[8]^ they are commonly used as descriptors to proxy electrocatalytic activities.[^9^, ^10^] The adsorption energies of these reactants are also linearly correlated with adsorption energies of the central atoms of these reactants.^[11]^ Therefore, CO_2_RR can be proxied using C‐adsorption energies,^[7]^ HOR and HER can be proxied using H‐adsorption energies,^[6]^ and ORR and OER can be proxied using O‐adsorption energies.[^4^, ^5^] According the Sabatier principle, a moderate atomic adsorption energy is necessary to obtain optimal electrochemical activities.[^12^, ^13^] Currently, the electrocatalysts with the highest activities are monometallic noble metals (e. g. Pt, Ru, Ir), but these are known to be expensive and scarce.^[14]^ Thus, electrocatalysts with non‐critical raw materials or non‐noble metals must be explored to achieve commercial profitability.

Much attention has been focused on constructing transition metal (TM) alloy electrocatalysts to reduce the amount of noble metals by introducing more abundant metals. Since TMs have more localized d‐states than their s‐ and p‐states, and electrons can easily participate in charge transfer processes, TM alloys can be tailor‐made based on the properties of the individual atoms. Bimetallic alloys are an example that balances these performance and scarcity concerns by combining TMs – typically a noble metal and a non‐noble metal.^[15]^ The same principles also apply to TM derivatives such as surface alloys,^[16]^ single atom catalysts,^[17]^ TM phosphides,^[18]^ and high‐entropy alloys.^[19]^ Density Functional Theory (DFT) is a computational technique based on quantum mechanics that utilizes the electron density of a system to optimize system structures and calculate adsorption energies.^[20]^ By using DFT to calculate the adsorption energies of molecules on bimetallic alloys, high‐throughput screening across large amounts of electrocatalyst candidates could be conducted. However, basing catalyst screening decisions solely on hundreds or thousands of DFT calculations would take weeks to months of calculation time, depending on the available computational resources. With the overwhelming volume of candidate materials, material screening approaches that are practical for big data must be applied.^[21]^

When coupled with DFT, Machine Learning (ML) is used to aid high‐throughput catalyst discovery, selection, and development.^[22]^ Using ML predictive models, large datasets containing quantitative properties of materials (features) are collected and output parameter values are estimated through regression. ML‐assisted DFT studies have been conducted to predict electrocatalyst properties such as adsorption energy^[23]^ and dopant segregation energy.^[24]^ The performance of these models is dependent on their features, which must be invariable, unique, compact, and computationally inexpensive.^[25]^ Since countless features are used to predict adsorption energies, there is no consensus on feature classification. One such example is shown in Table 1.^[26]^ The definitions of these features are discussed further in the Supporting Section S2.1.

**Table 1:** Features for adsorption energy prediction used in this study.

Feature Groups	Examples of Features
	
Electronic (based on electronic structure)	d‐Band Center (DBC)
	d‐Band Width (DBW)
	d‐Band Filing (DBF)
	Density of States at Fermi Energy (DOS)
	Work Function (WF)
Elemental (properties based on atomic identity)	Electron Affinity (EA)
	Local Electronegativity (EN)
	Ionization Energy (IE)
	Valence Electrons (VE)
	Sublimation Energy (SE)
	Lattice Constant (LC)
	Molar Volume (MV)
Structural (based on lattice arrangement)	Ensemble Atom Count (EAC)
	Generalized Coordination Number (GCN)

These feature groups have varying computational costs and prediction accuracies, so recent studies use a varying combination of these groups. For instance, T. R. Wang et al. in 2020^[27]^ utilized elemental and structural properties from online databases while Tomacruz et al. in 2022^[28]^ used elemental, structural, and electronic properties from online databases and DFT calculations to predict atomic adsorption energies on transition metals. Predicting adsorption energy is not the only insight that can be attained from these regression models. Structure‐property‐performance (SPP) relationships can be deciphered, which can help researchers engineer materials with optimized properties.^[22]^

Black‐box regression models such as neural networks and random forests provide a higher level of accuracy in exchange for a lower clarity on the SPP relationships in the models. Gray‐box interpretation methods are post‐hoc analysis techniques that address this trade‐off, because they explain the effects and importances of each feature after the black‐box model has completed its regression.^[29]^ Some of these gray‐box methods are model‐specific such as the extremely randomized trees method used by H. Li et al. in 2020,^[30]^ while others are model‐agnostic, which means that they are applicable to any regression model. Praveen & Comas‐Vives in 2020^[31]^ and Tomacruz et al. in 2022^[28]^ respectively used SHapley Additional exPlanations (SHAP) and Permutation Feature Importance (PFI) to identify the features that have contributed the most in atomic adsorption energy prediction. Since applying an interpretable ML–DFT approach has had limited exploration, the relationships of the previously mentioned features with atomic adsorption behavior on bimetallic TM alloys require deeper investigation.

This work implemented an interpretable ML–DFT approach to predict the adsorption energies of single‐atom adsorbates and display SPP relationships on monometallic and bimetallic transition metal surfaces.[^15^, ^32^] First, property datasets were constructed by collecting and generating feature data. Elemental properties of surfaces came from online databases, and structural and electronic properties were attained from site and DFT calculations. Then, atomic adsorption energies were predicted using fine‐tuned ML regression models. Finally, the contributions of each feature were evaluated using model‐agnostic interpretation methods. It should be noted that the study was only limited to fcc(111) surfaces and alloys with a 1 : 1 mixing ratio, and was limited to investigating SPP relationships of the TM surface. With this approach, we not only discover the accurate regression models for this dataset, but also evaluate the consistency of these models with theoretical concepts.

## Results and Discussion

### Exploratory Data Analysis from Property Datasets

In this section, we discuss the properties and trends behind the 426 transition metals and alloys obtained from Catalysis‐hub.org,[^15^, ^29^] 1393 C‐adsorption energies, 1897 H‐adsorption energies, and 1533 O‐adsorption energies were obtained as data points. The fourteen features listed in Table 1 were collected for each data point. DFT calculations were conducted to obtain the properties for electronic features, while online databases and site calculations are the sources for elemental and structural properties. More details in the DFT calculations are found below under Computational Methods, and references behind dataset construction are elaborated in Supporting Section S2. The subsections below display the theoretical trends observed from our datasets.

### d‐Band Model

**Figure 1:**
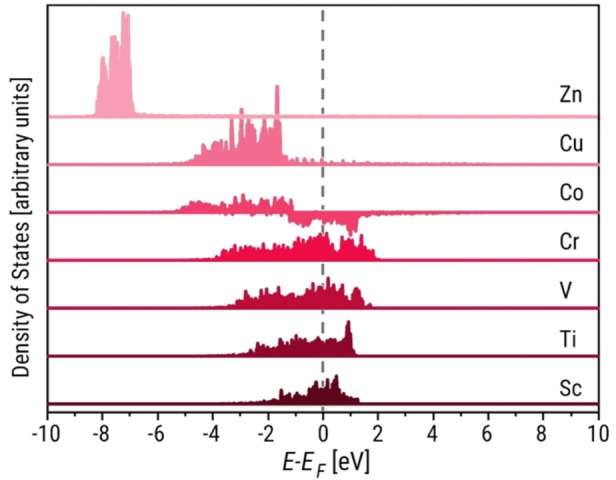
d‐band structures across 3d TM surfaces represented by PDOSs.

One popular relationship in these features is rooted in electronic theory – the d‐band model. The states from the d‐orbitals are more dense in certain energy ranges than states in than s‐ and p‐orbitals. In addition, d‐electrons do not provide a uniform contribution to atomic bonding, unlike sp‐electrons, and must therefore present relevant bonding insights. The d‐band model quantifies this by treating the d‐band center as a descriptor of the adsorption energy of an atom on a TM surface because it correlates with the position of the upper d‐band edge. Note that a decrease in the number of valence electrons is correlated with a positive shift in the d‐band structure, which indicates an increase of unoccupied states (i. e., the states above the Fermi energy). The anti‐bonding states of the surface and the adsorbate are then partially filled and thus adsorption is stronger (i. e., more negative adsorption energy).^[8]^ This model is corroborated by the electronic structures from DFT calculations in Figure 1, as the TM d‐band structures represented by their projected densities of states (PDOS) shift to the right when the number of valence electrons decreases. This results in stronger atomic adsorption energies, as discussed in Supporting Section S3.1.

### Friedel Model

**Figure 2:**
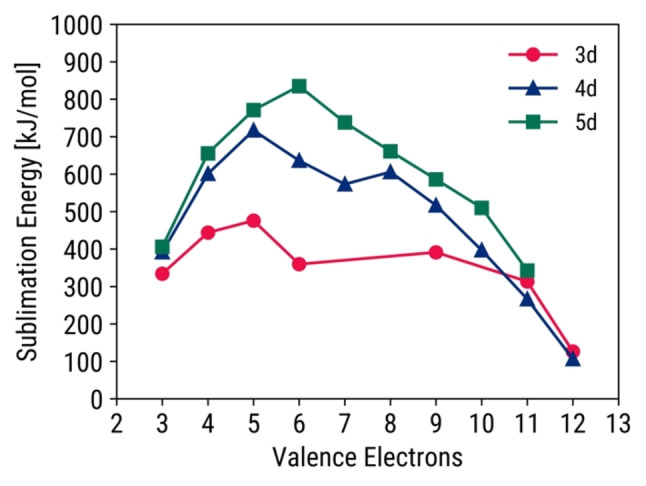
Parabolic or “double‐hump” plots between sublimation energy and number of valence electrons across 3d, 4d, and 5d TM surfaces.

The Friedel model is another electronic theory, where sublimation energy and the number of valence electrons demonstrate a parabolic or “double‐hump” behavior. Due to Hund's rule, unpaired electrons initially fill the d‐orbitals which increases binding and cohesive energy. Once all the d‐orbitals have unpaired electrons, the antibonding d‐states are then filled which decreases binding and cohesive energy.^[33]^ It should also be noted that half‐filled electron subshells have additional stability and thus decrease binding and cohesive energy.^[34]^ Therefore, it is expected for TMs with three to four unpaired d‐electrons (i. e., five to six valence electrons) to have the highest cohesive energy. Since cohesive energy and sublimation energy are highly correlated, the latter also demonstrates the same parabolic behavior (Figure 2). Dips in the center are visible for 3d and 4d TMs due to the half‐filled d‐orbitals. Cohesive energy is also indirectly related to adsorption energy, so an indirect relationship between sublimation energy and adsorption energy is expected.^[34]^ This was found to be corroborated and discussed in Supporting Section S3.1, particularly for C‐adsorption.

### Limitations of d‐Band Model for Prediction

**Figure 3:**
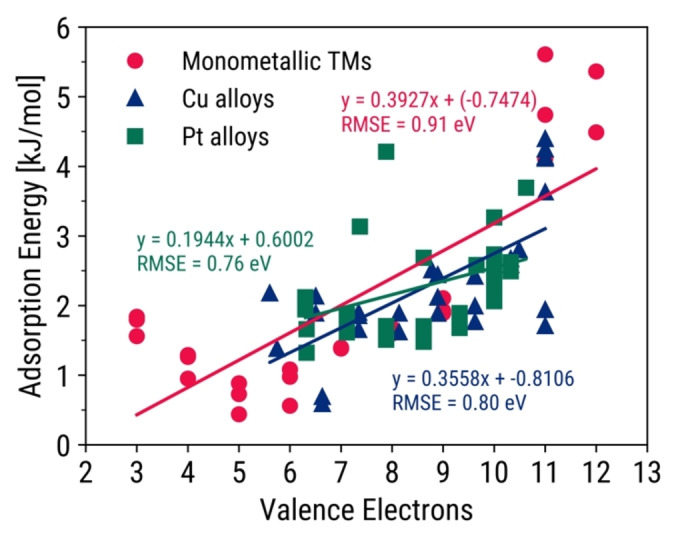
C‐adsorption energies and valence electrons of TMs, TM Cu‐alloys and TM−Pt alloys. Their best fit lines and RMSE values are also indicated.

Despite the presence of theoretical trends in the datasets, they could not be relied on as individual descriptors for atomic adsorption energy prediction. The linear plots in Figure 3 contain several deviations, mostly from alloys with elements containing d^1^, d^2^, d^5^, d^9^, or d^10^ electrons. Metals with the latter two subshells could not be treated as outliers because noble metals are highly relevant for electrochemical reactions. The same observations are obtained with other descriptors such as sublimation energy, d‐band center, and the d‐band upper edge (DBUE). The d‐band upper edge is a modification of the d‐band model that incorporates both d‐band center and d‐band width, as seen in Equation 1.

(1) $$DBUE=DBC+\frac{DBW}{2}$$

The linear scaling relation plots of the descriptors mentioned above are discussed in Supporting Section S3.2, and are shown to have higher root mean square error (RMSE) values than those from ML models in literature,[^27^, ^28^] particularly for C‐ and O‐adsorption. These findings present an opportunity for ML models to provide more accurate predictions when given these properties.

### Feature Engineering Using Feature Selection

Reducing the number of features through feature selection is necessary to speed up calculation time while minimizing noise from redundant features. The fourteen features in each adsorption dataset are pruned using Ward's linkage, since this agglomerative method is practical for compact clusters with features that are highly correlated and equivalent in size.^[35]^ First, the distances between two features are defined using Equation (2), where $\rho_{i,j}$ is the Spearman rank correlation coefficient between features i and j . A square matrix of these distances is then condensed to a vector v via binomial expansion, as seen in Equation 3.^[36]^

(2) $$d_{i,j}=1-\left|\rho_{i,j}\right|$$

(3) $$v\left[\frac{n!}{(n-2)!2!}-\frac{\left(n-i\right)!}{\left(n-2-i\right)!2!}+\left(j-i-1\right)\right]=d_{i,j}$$

From here, a distance $d_{i,j}$ is calculated. Finally, iterative loops occur on the condensed matrix where smaller clusters with minimal distances (or marginal variance increase) are agglomerated, gradually forming a dendrogram of clusters from the bottom to the top.^[37]^ As a result, highly correlated features are clustered at the bottom of the dendrogram. A cut‐off distance of 0.5 was set because information loss increases as the distance also increases.^[38]^

**Figure 4:**
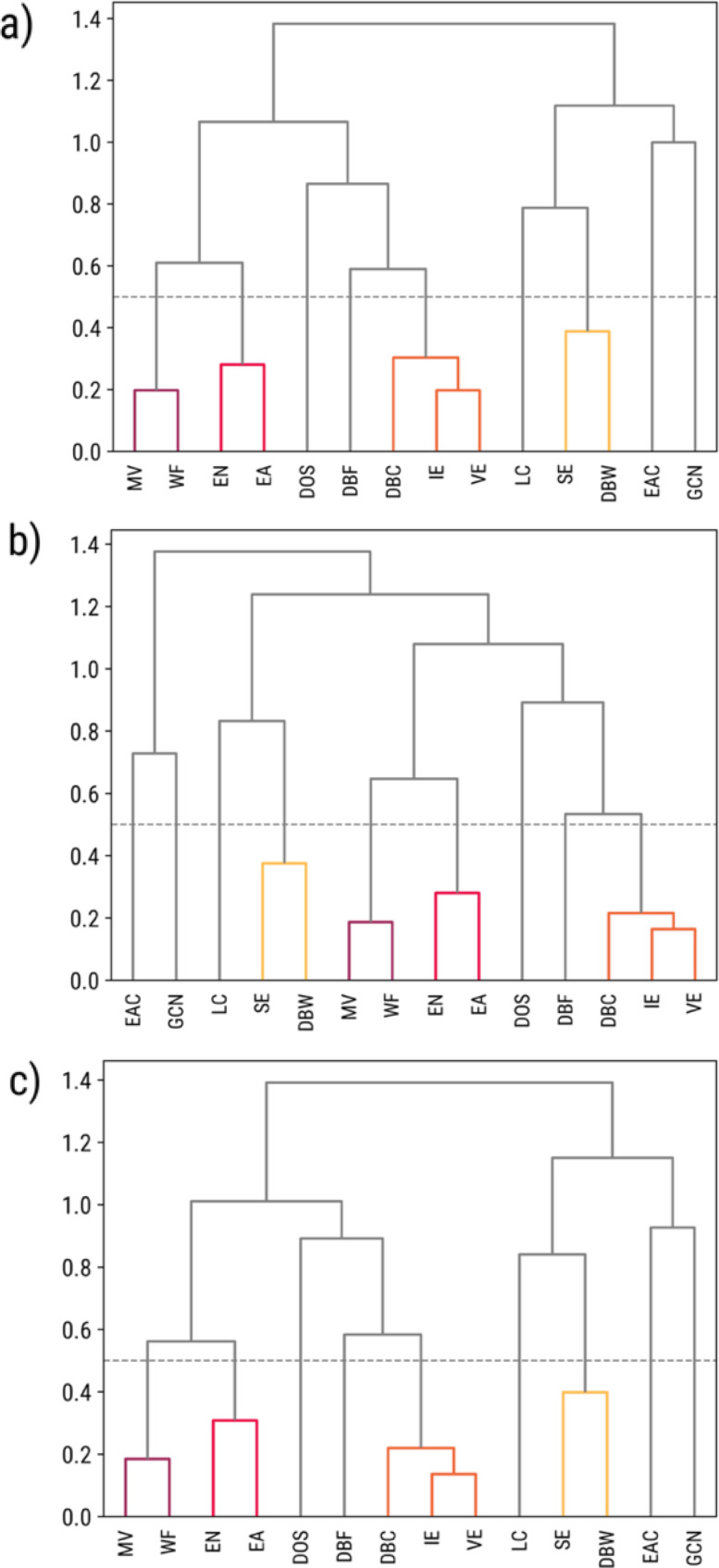
Dendrograms representing the nine clusters observed in (**a**) C‐, (**b**) H‐, and (**c**) O‐adsorption given a threshold distance of 0.5. The colored clusters indicate clusters of *n* highly‐correlated features.

With a distance threshold of 0.5, all fourteen features from all property datasets were simplified to the same nine clusters (Figure 4). Four of these clusters were formed from multiple features. These are namely: the MV‐WF cluster, the SE‐DBW cluster, the EN‐EA cluster, and the DBC‐IE‐VE cluster. These clusters have highly correlated features because of previously discovered physics‐based relationships,[^17^, ^39^, ^40^, ^41^, ^42^] which are discussed in Supporting Section S4.

Under this threshold, each cluster with *n* highly correlated features was simplified by choosing the feature with the highest Spearman rank correlation coefficient with adsorption energy, while the other *n‐1* features were removed. For example, H‐adsorption energy has a higher correlation with the d‐band center than with ionization energy or valence electrons. Therefore, the DBC‐IE‐VE cluster is reduced to the DBC feature in the H‐adsorption dataset. Comprehensive details are discussed in Supporting Section S4.

### Atomic Adsorption Energy Prediction Using ML Regression Models

The reduced datasets were then sent to hyperparameter optimization and model testing. The selected hyperparameters from the best‐performing model of each regression algorithm are listed in Table 2. The RMSEs of these models are listed in Table 3. Parity plots of all three regression algorithms on the three adsorption prediction datasets are shown in Supporting Section 5.2.

**Table 2:** Optimized hyperparameter values in different atomic adsorption energy prediction models.

ML Model	Hyperparameter	Optimized Values		
		C‐adsorption	H‐adsorption	O‐adsorption
Random Forest Regression (RFR)	Number of Trees	150	100	100
	Maximum Tree Depth	20	20	15
	Number of Feature Inputs	sqrt	sqrt	sqrt
Gaussian Process Regression (GPR)	Alpha	0.1	1×10^−10^	0.01
	Kernel	Matern+Dot Product^[a]^	RQ+Matern^[b]^	RQ+Matern^[c]^
Artificial Neural Network (ANN)	Batch Size	10	10	15
	Neurons in second hidden layer	20	15	15
	Neurons in third hidden layer	10	10	10
	Learning Rate	0.001	0.001	0.01

[a] ConstantKernel(8.8209)*Matern(length scale=6.14, nu=1.5)+ConstantKernel(0.019881)*DotProduct(sigma_0=29.3)+WhiteKernel(2.76×10^−08^); [b] ConstantKernel(0.451584)*RationalQuadratic(alpha=1e+05, length scale=2.36)+ConstantKernel(3.3124)*Matern(length scale=8.98, nu=1.5)+WhiteKernel(0.0222); [c] ConstantKernel(8.0656)*RadialQuadratic(alpha=0.017, length scale=3.25)+ConstantKernel(11.4921)*Matern(length scale=9.9, nu=1.5)+WhiteKernel(0.00317)

**Table 3:** ML regression model accuracies after model training and testing.

ML Model	C‐adsorption		H‐adsorption		O‐adsorption	
	Train RMSE	Test RMSE	Train RMSE	Test RMSE	Train RMSE	Test RMSE
RFR	0.163	0.356	0.085	0.178	0.164	0.351
GPR	0.206	0.361	0.122	0.179	0.046	0.375
ANN	0.364	0.435	0.178	0.221	0.377	0.429

**Figure 5:**
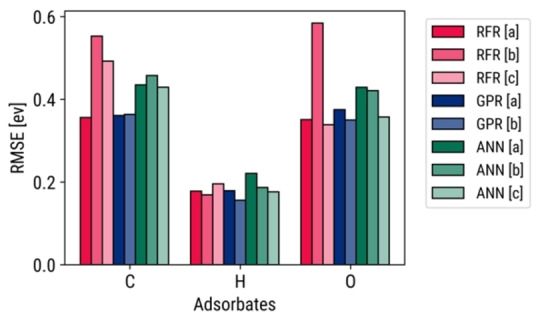
Benchmaring RMSEs of this work [a] against similarly constructed models ([b]: Tomacruz et al., 2022,^[28]^ [c]: T. R. Wang et al.^[27]^)

These accuracies are compared to those from similar studies that have used Catalysis‐hub.org as their reference database for atomic adsorption energies, as seen in Figure 5. The trained models demonstrate a comparable RMSE to those from Tomacruz et al. (2022)^[28]^ and T. R. Wang et al. (2020).^[27]^ This is despite the fact that the datasets from only Tomacruz et al.^[28]^ only contained fewer data points and less noise, while those from T. R. Wang et al.^[27]^ only contained the most stable adsorption energies for all surfaces. Filtering only the most stable adsorption sites is a common step for data cleaning,[^27^, ^43^] but ML–DFT studies that do not screen the most stable sites still obtain RMSEs of up to 0.4 eV.^[44]^ The model accuracies of this work could be improved in future work by including only the most stable adsorption sites in the datasets and adding alloys with different mixing ratios. Details on benchmarking are also found in Supporting Section 5.2.

### Feature Contributions from Interpretation Methods

**Figure 6:**
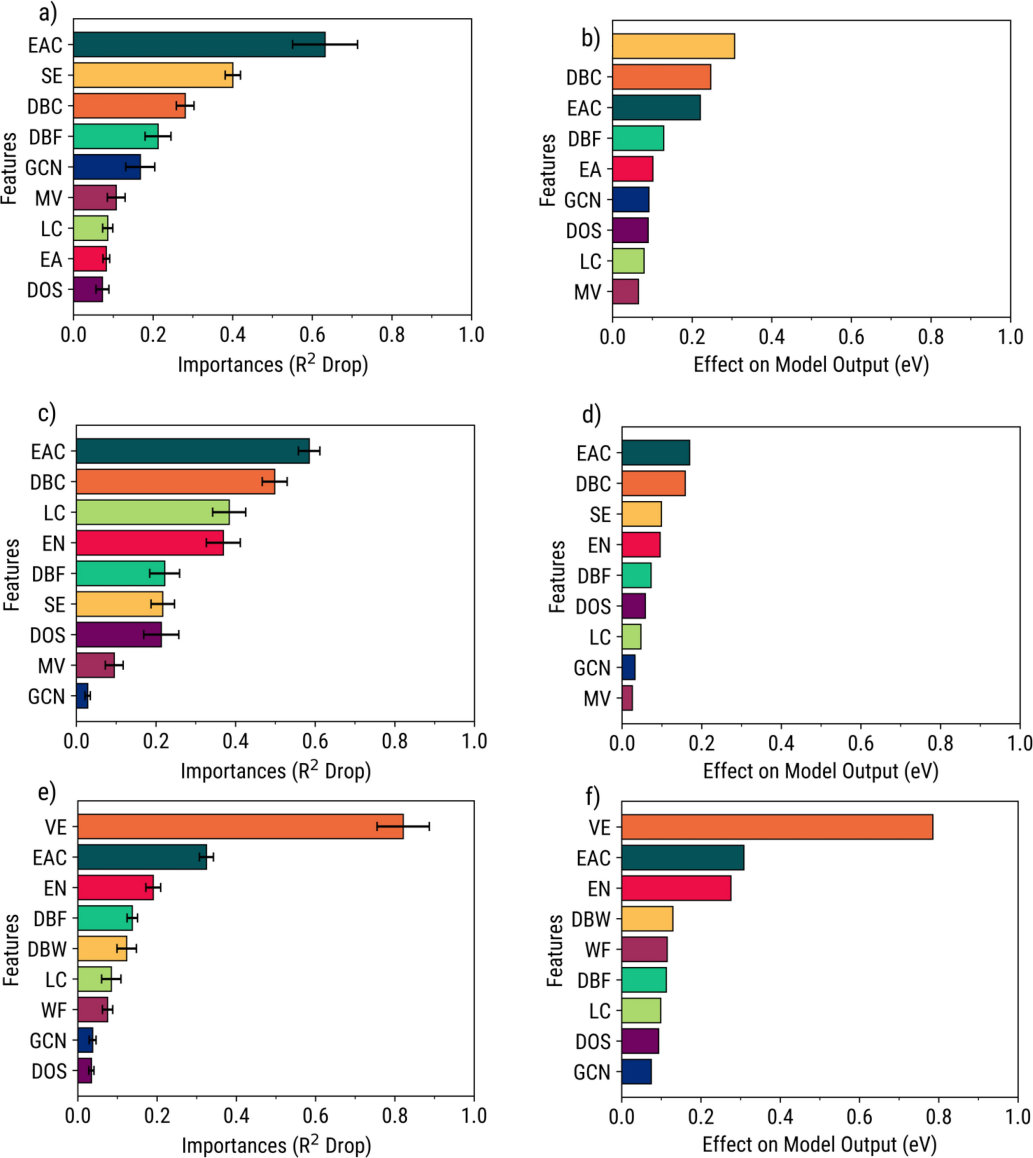
Feature importances in GPR models using interpretation methods: PFI [left] on C‐ (**a**), H‐ (**c**), and O‐ (**e**) property datasets, and SHAP [right] on C‐ (**b**), H‐ (**d**), and O‐ (**f**) property datasets.

Interpretation methods PFI and SHAP were used to measure the global feature importances, which are shown in Figures 6, and Supporting Section S6. Although the feature rankings may shuffle between the two methods, the top features in PFI are also those in SHAP. Therefore, these two features in each dataset satisfy the “largest contribution” criteria for both definitions. The same behavior was observed across most ML models (Table 4).

**Table 4:** The top two features or feature clusters with the highest importances according to PFI and SHAP. Note that some are clusters from Ward's linkage.

Model	*C	*H	*O
RFR	EAC^[PFI]^ or SE/DBW^[SHAP]^	EAC	VE‐IE‐DBC
	DBC‐VE‐IE	DBC‐VE‐IE	EAC
GPR	EAC^[PFI]^ or DBC‐VE‐IE^[SHAP]^	EAC	VE‐IE‐DBC
	SE/DBW	DBC‐VE‐IE	EAC
ANN	EAC	DBC‐VE‐IE	VE‐IE‐DBC
	SE/DBW	EAC	EAC

Out of the nine different features or feature clusters, only three were observed to provide the highest contributions consistently. First, the importance of the ensemble atom count could not be understated since most atomic adsorbates were generally more stable at higher‐fold adsorption sites.^[45]^ This insight was obtained since all adsorption sites were considered, as opposed to screening only the most stable adsorption sites. Second, the value of the d‐band model is shown through the high importance of the cluster containing the d‐band center, valence electrons, and the ionization energy. Last, the Friedel model was shown to be important for C‐adsorption, as indicated by the high importance of the cluster containing sublimation energy and d‐band width.

It should also be stated that these top features are not highly correlated with each other, as shown by the larger distances between the feature clusters in Figures 4a–c and Supporting Section S4, suggesting that the contributed information of each feature or feature cluster is not redundant. Furthermore, it is notable that these top features belong to the site atomic and site structural‐electronic subgroups, suggesting that characterizing the unique identities of each adsorption site provides more value in predicting adsorption energy than describing the properties of the entire surface. It should be stressed though that these insights do not universally apply to all possible combinations of features, as different dataset compositions will result in different top features.

**Figure 7:**
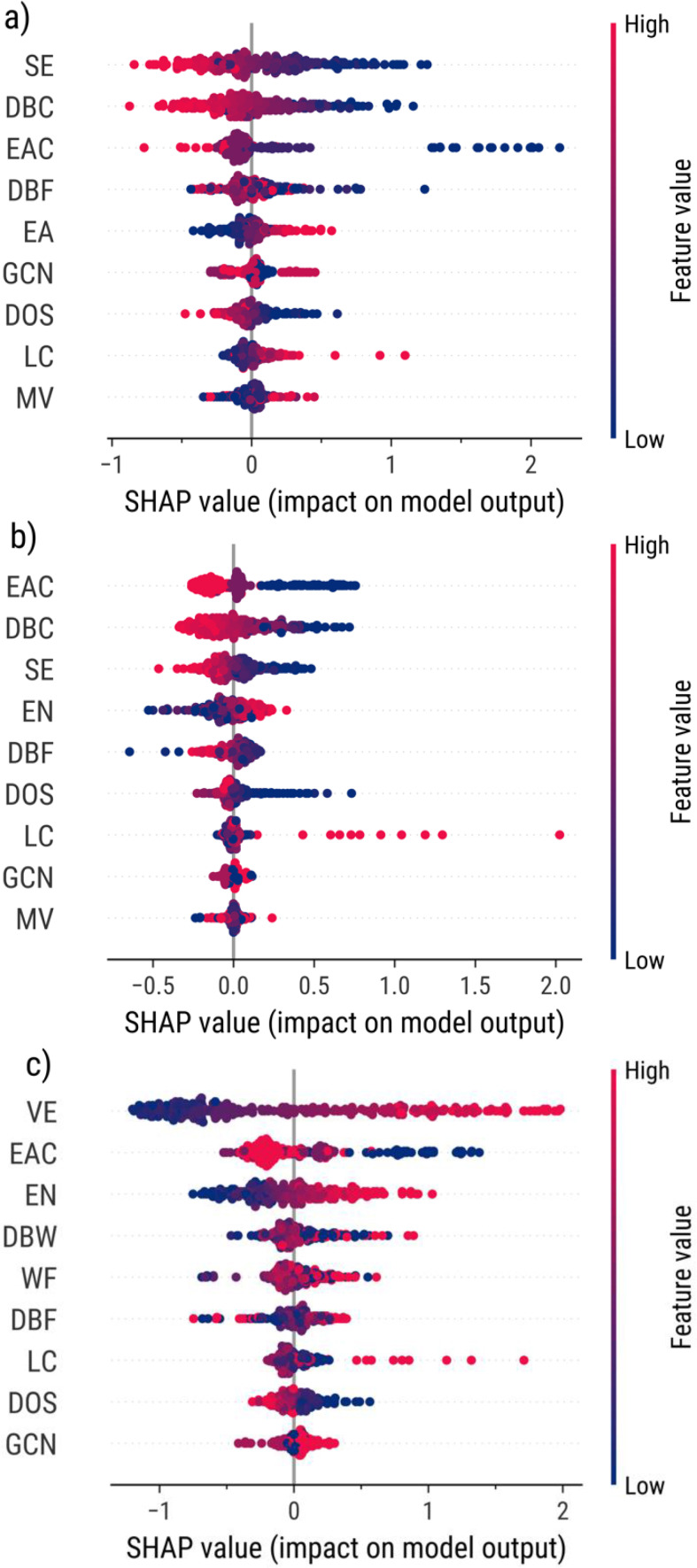
Local feature effect plots on (**a**) C‐, (**b**) H‐, and (**c**) O‐adsorption energy predictions with GPR model using SHAP.

In addition to the results from global feature importances, Figure 7 illustrates the direct and indirect relationships of features with adsorption energy prediction and their consistency to electronic theories. For example, all models show that a high‐value d‐band center corresponds to a more negative model output and a stronger adsorption energy – consistent with the d‐band model. The same is observed for ensemble atom count and the Friedel model. Thus, this shows that these regression models can accurately predict atomic adsorption energies while following the SPP relationships of monometallic and bimetallic TMs.

## Conclusions

In this work, the adsorption energies of carbon, hydrogen, and oxygen on transition metals and bimetallic alloys were predicted using a combined ML–DFT approach. First, adsorption energy prediction databases were constructed from online databases, site calculations, and DFT calculations on adsorbate‐free TM surfaces. Second, trends from theoretical models such as the d‐band and Friedel models were found in the adsorbate datasets. Third, GPR and RFR were found to exercise the highest accuracies in adsorption energy prediction after hyperparameter optimization and model training and are comparative to literature. Last, the results from interpretation methods suggest that the most globally important features and prevalent local trends in the ML models were consistent with the d‐band model, the Friedel model, and the preference of atomic adsorbates to adsorption sites with a higher ensemble atomic count.

Future work can be dedicated to the development of this methodology. On the data collection and DFT side, this methodology can be expanded to materials with more mixing ratios (e. g., 1 : 3, 3 : 1), more complex metals (e. g., trimetallic alloys, high entropy alloys), as well as incorporating more complex electronic properties. Adsorbate properties can also be included in future datasets, so that information regarding SPP relationships of adsorbates can be explored. On the ML side, feature engineering could be improved by exploring non‐linear dimensionality reduction methods such as kernel principal component analysis. More regression methods such as support vector regression and extreme gradient boosting can be explored, and the predictive models should also handle data at various train‐test splits (e. g., 60 %–40 %, 20 %–80 %). Lastly, additional feature effect explanations from SHAP could be extracted from specific alloys, particularly in non‐noble and non‐critical metals.

Aside from these methodology improvements, this ML–DFT approach may also be extended to predict other electrocatalytic data and descriptors (e. g., overpotential, turnover frequency), as well as experimental data. An overall dataset predicting the adsorption energies of all adsorbates could also be constructed. Finally, this approach can act as the foundation of an iterative cycle of adsorption energy predictions and DFT calculations to screen electrocatalyst materials that are not found in online databases. Overall, this work is a successful proof of concept for using ML models on a comprehensive transition metal search space with interpretation methods to explain structure‐property‐performance insights and assure material scientists that these models are consistent with underlying physics concepts.

## Computational Methods

### Density Functional Theory‐Based Calculations

DFT calculations were conducted using Quantum Espresso v6.6.^[46]^ The Perdew‐Burke‐Ernzerhof (PBE) functional was used with Bayesian error functional with van der Waals correlation (BEEF‐vdW),^[47]^ as this accounts for vdW interactions. Although systematic errors are still present in BEEF‐vdW compared to total energy methods, the functional is more accurate in calculating adsorption energies than its contemporaries.^[48]^ We utilize ultra‐soft pseudopotentials, as these converge at low energy cutoffs, resulting in quicker self‐consistent field (SCF) iterations.^[20]^

The atomic coordinates for 26 monometallic and 400 bimetallic transition metal surfaces were all obtained from Catalysis‐hub.org.^[15]^ The structures and identities of these alloys are shown in Supporting Section S1. These structures were assumed to be optimized, so relaxation calculations were not conducted. Instead, SCF calculations were conducted at an energy convergence threshold of 1.36×10^−4^ eV. Convergence tests resulted in a 12×12×1 Monkhorst‐Pack k‐point mesh, a 750 eV kinetic energy cutoff for wavefunctions, and a 7500 eV kinetic energy cutoff for charge density. Mirroring the assumptions used in Mamun et al. (2019),^[15]^ nonpolarized calculations were performed on all surfaces except for metals and alloys with Mn, Fe, Co, and Ni, which had spin‐polarized calculations with starting magnetizations of 0.333, 0.375, 0.706, and 0.5, respectively. A vacuum space of 20 Å was inserted along the direction perpendicular to the surface normal to simulate an infinite metal slab across the x‐axis and y‐axis but finite in the z‐axis. In addition, a dipole correction was also applied to the surface to cancel the electric field across the slabs due to the slab's periodicity.

The same parameters were also used for post‐processing (PP) and electronic structure calculations. An electrostatic curve was generated from PP calculations to obtain the work function of the surface, which is the difference between the vacuum energy and the Fermi energy.^[49]^ The densities of states (DOS) of transitional metal surfaces at the Fermi energy were calculated to display the contributions of both the sp‐ and d‐bands at the Fermi level,^[50]^ while the partial densities of states (PDOS) were calculated to obtain the d‐band center (Equation (4)), d‐band width (Equation (5)), and d‐band filling (Equation (6)) to provide insights into the d‐band structure of the surface.^[51]^

(4) $$DBC=\frac{\int_{-\infty }^{\infty }n\left(E\right)\cdot \left(E-E_{F}\right)}{\int_{-\infty }^{\infty }n\left(E\right)}$$

(5) $$DBW={\left(\frac{\int_{-\infty }^{\infty }n\left(E\right)\cdot [E-E_{F}-DBC{]}^{2}}{\int_{-\infty }^{\infty }n\left(E\right)}\right)}^{\frac{1}{2}}$$

(6) $$DBF=\frac{\int_{-\infty }^{E_{f}}n\left(E\right)}{\int_{-\infty }^{\infty }n\left(E\right)}$$

Where E describes the energy level, $n\left(E\right)$ is the number of states at that level, and $E_{F}$ is the fermi energy of the surface. These equations were based on the semi‐elliptical approach for d‐band properties.^[51]^

### Adsorption Dataset Preparation and Data Treatment

**Figure 8:**
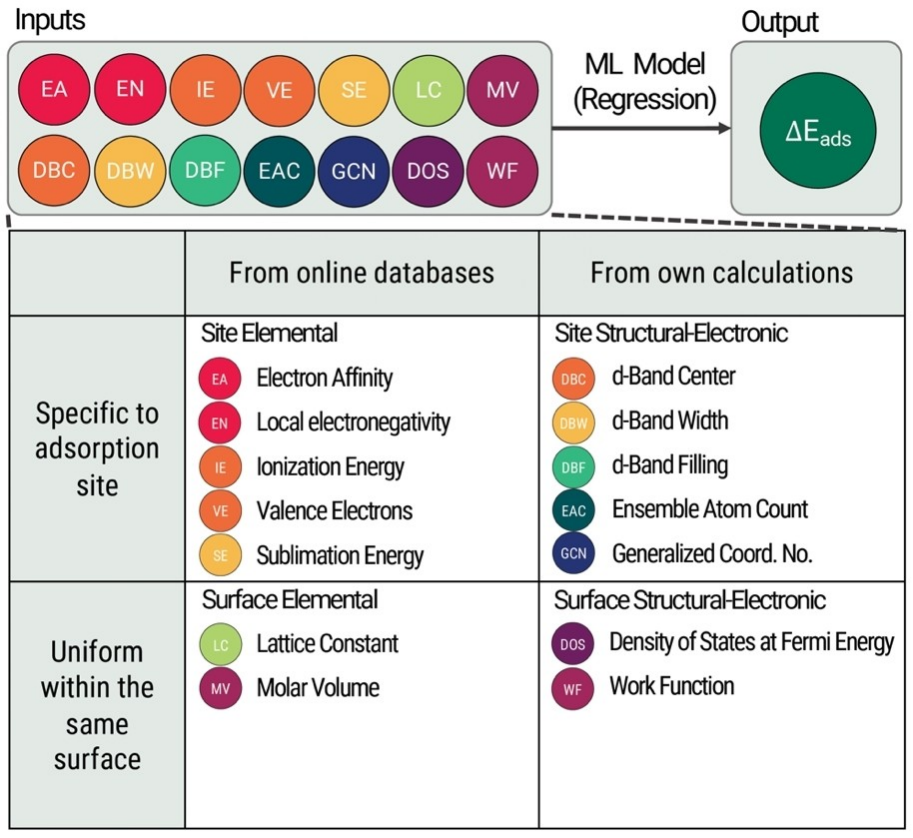
Breakdown of atomic adsorption energy prediction dataset features and their categories, which are classified based on ease of access and if there are localities present.

Three datasets were prepared for regression modeling – one for each adsorbate (C, H, and O). Each dataset (Figure 8) was initially composed of fourteen features and was categorized based on their accessibility and dependence on surface structure. The atomic adsorption energies of C, H, and O on the 426 transition metal surfaces were obtained from Catalysis‐hub.org.[^15^, ^32^]

Elemental features describe the atomic identity and composition of the surface atoms, while structural features represent the arrangement of atomic coordinates, and electronic features explain the orbital interactions between atoms and surfaces. Due to the small supercell size, the adsorption site is limited to the atoms that are immediately adjacent to the adsorbate. The different adsorption sites are listed in Supporting Section S2.2. Elemental, structural, and electronic features have been used in existing machine learning studies for prediction[^21^, ^22^, ^25^, ^27^, ^50^, ^52^] since there are physics‐based trends that relate these properties to adsorption energy.[^8^, ^34^, ^45^] Explanations as to how these features were calculated were explained in the previous subsections and Supporting Section S2.1. Exploratory data analysis was conducted to show the individual relationships of each feature with adsorption energy, also in the Supporting Information.

Feature selection was conducted using Ward's linkage, which was discussed in the section above. Since all the features in each dataset were assumed to be numerical and continuous, each dataset was normalized with unit variance and zero mean, then fitted to three ML regression methods (Advantages and disadvantages are in Supporting Section S5.1).

### Interpretable Machine Learning Implementation

The models were trained on ten randomized and different train‐test splits of 80 %:20 %, where the hyperparameters of each model were optimized. Through a five‐fold grid‐search cross validation, the model with the lowest RMSE and their hyperparameters were saved. Further details regarding hyperparameter optimization are in Supporting Section S5.1. The RMSEs of these models were the compared against similar ML models constructed by studies that used Catalysis‐hub.org as their database for adsorption energies, as seen in Supporting Section S5.2.

Model‐agnostic interpretation methods were used on the saved models. Global feature contributions and local feature effects were obtained from Permutation Feature Importance (PFI) and SHapley Additive exPlanations (SHAP). PFI calculates the global feature importances by corrupting one feature within the dataset and observing the R^2^ drop. The features with the highest drops in accuracy are the features with the highest contributions during prediction.^[53]^ Meanwhile, the SHAP method applies cooperative game theory to identify the average marginal contributions of each feature to the predictive model. Unlike PFI, marginal contributions are calculated across all possible subsets of features, which allows interaction effects to be accounted. As a result, SHAP provides both global feature effects through feature rankings, and local feature effects by displaying directional relationships between features and their marginal contribution towards adsorption energy prediction.^[54]^ A more comprehensive comparison of these methods is provided in the Supporting Section S6.

## Supporting Information Summary

The Supporting Information contains a discussion on data collection and generation for property datasets, a discussion on the highly correlated clusters from Ward's Linkage, details on hyperparameter optimization, a comparison of ML regression methods, a discussion on PFI and SHAP, and benchmarking results with those from related literature.[^4^, ^6^, ^7^, ^8^, ^15^, ^20^, ^32^, ^36^, ^38^, ^39^, ^40^, ^41^, ^42^, ^43^, ^46^, ^51^, ^53^, ^54^, ^55^, ^56^, ^57^, ^58^, ^59^, ^60^, ^61^, ^62^, ^63^, ^64^, ^65^, ^66^, ^67^, ^68^, ^69^, ^70^, ^71^, ^72^, ^73^]

The data and code for the machine learning techniques used are uploaded in: https://github.com/GoranTomacruz/ML–DFT‐Bimetallics.^[73]^

## Conflict of Interests

The authors declare no conflict of interest.

1

## Supporting information

As a service to our authors and readers, this journal provides supporting information supplied by the authors. Such materials are peer reviewed and may be re‐organized for online delivery, but are not copy‐edited or typeset. Technical support issues arising from supporting information (other than missing files) should be addressed to the authors.

Supporting Information

## Data Availability

The data that support the findings of this study are openly available in ML‐DFT Bimetallics at https://github.com/GoranTomacruz/ML‐DFT‐Bimetallics, reference number 73.
