# Supplementary material for: Atomic Adsorption Energies Prediction on Bimetallic Transition Metal Surfaces Using an Interpretable Machine Learning‐Accelerated Density Functional Theory Approach
Source: ChemistryOpen. 2025 Jan 29;14(4):e202400124. doi: 10.1002/open.202400124 (PMC11973507; doi:10.1002/open.202400124)
Supplement: Supplementary file 1 — Supporting Information [file OPEN-14-e202400124-s001.pdf]

ChemistryOpen

Supporting Information

Atomic Adsorption Energies Prediction on Bimetallic Transition Metal Surfaces Using an Interpretable Machine Learning-Accelerated Density Functional Theory Approach

Jan Goran T. Tomacruz, Michael T. Castro, Miguel Francisco M. Remolona, Allan Abraham B. Padama, and Joey D. Ocon*

ChemistryOpen
Supporting Information

Prediction of Atomic Adsorption Energies on Bimetallic Transition Metal Surfaces and Model Interpretation using an Interpretable Machine Learning-accelerated Density Functional Theory (ML-DFT) Approach

Jan Goran T. Tomacruz, Michael T. Castro, Miguel Francisco M. Remolona, Allan Abraham B. Padama, and Joey D. Ocon*

S1. Density Functional Theory-based Calculations

S1.1. Surface Selection and Adsorption Energies from Online Database

The transition metal surfaces from Catalysis-hub.org[32] and their atomic coordinates were found in this Materials Cloud link (https://archive.materialscloud.org/record/2019.0015/v1). The alloy structures were extracted from 4,017 Quantum ESPRESSO v5.1 output files from the calculations of Mamun et al.[15] A Python script was used to meticulously read each output file and identify composition and alloy mixing ratio. The output files were then manually filtered to remove surfaces that included earth metals, metalloids, and gas molecules, as well as alloys with different adsorption coverages and mixing ratios aside from 1:1. The optimized atomic coordinates of the selected surfaces were acquired from this script and placed in .txt files.

S1.2. Self-consistent Field Calculations

Then, more Python scripts were used to construct Quantum EPRESSO v6.6 input files from given structures. Each clean (or adsorbate-free) transition metal surface was modeled as a three-layered slab with four atoms per layer, as seen in Figure S1a. The atoms of the optimized structures are not all equidistant from each other, as the topmost layer of each slab was unconstrained by Mamun et al. (2019)[15] during relaxation. This is appropriate since the coverage of all atomic adsorbates across the surface is 0.25 Monolayer, or one adsorbate for every four surface atoms. The total energies of clean transition metal surfaces were calculated using single-point self-consistent field (SCF) calculations (pw.x in Quantum ESPRESSO). Most of the calculation conditions have already been discussed in the "Computational Methods" section from the main text. As a result, only 426 surfaces remained in the adsorption datasets. The TM and bimetallic TM alloys used in this study are listed in Figure S1b.

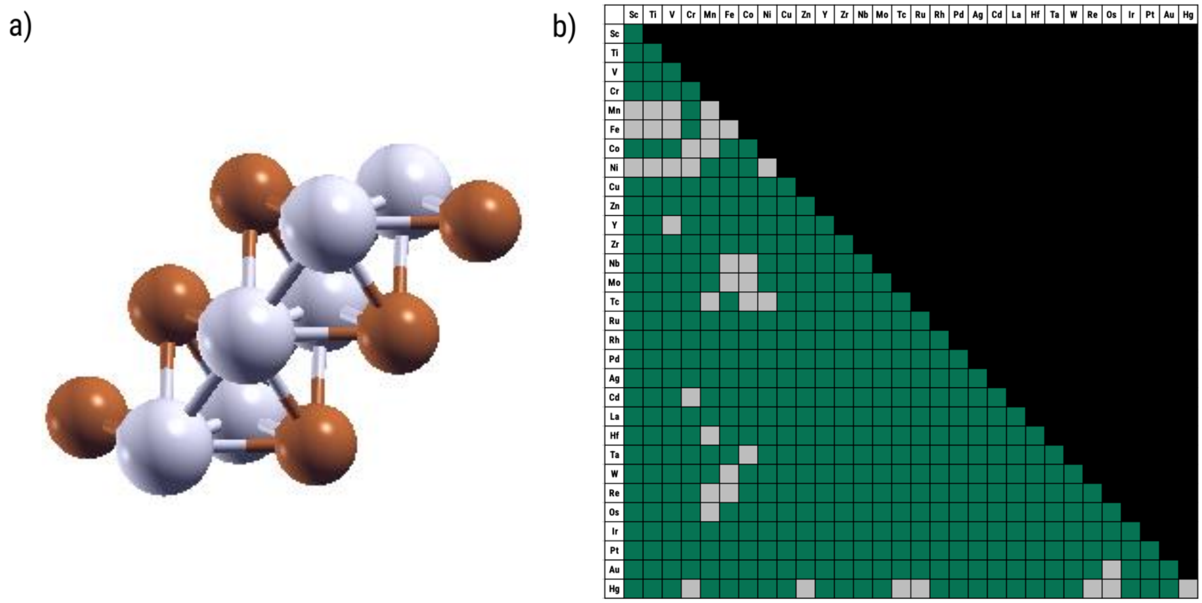

Figure S1. Diagrams of a) extracted TM structures, and b) monometallic TMs and TM alloys in the scope of this study. Surfaces with adsorption energies and structural coordinates in green, while surfaces with neither in gray.

S1.3. Post-processing Calculations

After SCF calculations, Density of States (DOS) and Project Density of States (PDOS) calculations were conducted using the dos.x and projwfc.x operations of Quantum ESPRESSO, respectively. The DOS values at fermi energy represent the contributions of both the sp-band and the d-band at the Fermi level,[43] while the overall shape of the d-band states from PDOS were approximated as a semi-ellipsis, and characterized by the d-band center, d-band width, and d-band filling.[51]

The work function is defined as the difference between vacuum energy (E_{vac}) and the Fermi energy (E_F). To obtain E_{vac}, the planar average of the sum of the bare potential (local ionic potential) and the bare Hartree potential (electrostatic potential from electron charge density) must be obtained with respect to the z-axis.[55-56] This is accomplished by conducting post-processing (PP) calculations after SCF calculations. Since the slab molecule is located at the center of the cell, E_{vac} is equivalent to the energy at the bottom or top of the cell.

S1.4. Site Calculations

Aside from DFT, geometric calculations were also conducted to characterize each adsorption site to two structural features: ensemble atom count and generalized coordination number. The ensemble atom count was obtained by counting the number of surface atoms in the adsorption site, while the generalized coordination number was calculated by counting the second nearest neighbors (atoms adjacent to the second degree) of the adsorption site, and applying those to Equation S1.

$$GCN = \sum_{i=1}^{n_i} \frac{CN(i) * n_i}{CN_{max}} \tag{S1}$$

Where $CN(i)$ is the number of second-nearest neighbors for nearest neighbor i, and CN_{max} is maximum number of neighbors in an atom given a certain surface facet.[57]

S2. Dataset Construction

S2.1. Input Variables (Descriptors)

Three datasets were prepared for regression modeling – one for each adsorbate (C, H, and O). Each dataset was initially composed of fourteen features and can be categorized based on their accessibility and dependence on geometric positions. Table S1 lists their definitions and data sources. It should be noted that the adsorption ensemble is defined as the surface atoms in the adsorption site and describes the local substrate environment of the adsorbate. Although there are other studies in literature that also account for the nearest neighbor atoms in the local environment,[58-59] this study only limited the local environment to the adsorption ensemble because the high adsorbate coverage will cause interference in calculating electronic effects if nearest neighbor atoms were included.

Table S1. Definitions of features in adsorption energy prediction dataset.

Subgroup	Feature	Definition	Data Source
Site Elemental	Electron Affinity (EA)	Energy difference between ground-state of a gas-phase atom and the lowest state of the corresponding anion [60]	Handbook of Chemistry and Physics 102nd Edition (2021) [60]
	Ionization Energy (IE)	Minimum required energy to remove an electron from an isolated and gas-phase atom in its ground-state [60]	NIST: Atomic Spectra Database - Ionization Energies Form (2020) [61]
	Local electronegativity (EN)	(Pauling definition) Relative power of atoms at the adsorption site to attract electrons [60]	Handbook of Chemistry and Physics 102nd Edition (2021) [60]
	Sublimation Energy (SE)	Required energy to cause a solid to gas phase change [60]	WebElements (1993) [62]
	Valence Electrons (VE)	Number of electrons in the outermost shell [60]	Periodic Table
Surface Elemental	Molar Volume (MV)	Atomic weight multiplied by density [60]	WebElements (1993) [62]
	Lattice Constant (LC)	Length of the smallest edge in unit cell [60]	WebElements (1993) [62]
Site Structural-Electronic	d-Band Center (DBC)	Center of d-band energy states at the adsorption site [51]	DFT Calculations
	d-Band Width (DBW)	Width of d-band energy states at the adsorption site [51]	DFT Calculations
	d-Band Filling (DBF)	Filling of d-band energy states at the adsorption site [51]	DFT Calculations
	Ensemble Atom Count (EAC)	Number of atoms at the adsorption site [43]	Catalysis-hub.org [32]
	Generalized Coordination Number (GCN)	Weighted ensemble atom count based on first and second nearest neighbors [57]	Structural Calculations
Surface Structural-Electronic	Density of States at Fermi Energy (DOS)	Density of states present when the energy is at the Fermi level [43]	DFT Calculations
	Work Function (WF)	Required energy to remove electron from inside the surface [60]	DFT Calculations

S2.2. Output Variable (Adsorption Energy)

The Catalysis-hub API was also used to obtain the adsorption energies of carbon, hydrogen, and oxygen. The adsorption energies of the three adsorbates were calculated by Mamun et al. (2019)[15] based on Equations S2 to S4, where ΔE_i is the total energy of the system i after SCF calculations. It should be noted that although there are 426 structures present in this study, each surface has more than one adsorption site (the difference kinds of sites are listed in Table S2). These adsorption energies were then matched to their respective surface.

Table S2. List of adsorption sites in the atomic adsorption prediction datasets

Adsorption site	Ensemble atom count	Present in monometallic TMs or bimetallic TM alloys?
top|A	1	Both
bridge|A_A|A	2	Both
hollow|A_A_A|HCP	3	Monometallic
hollow|A_A_A|FCC	3	Monometallic
top|B	1	Bimetallic
bridge|A_A|B	2	Bimetallic
bridge|A_B|A	2	Bimetallic
bridge|A_B|B	2	Bimetallic
bridge|B_B|A	2	Bimetallic
bridge|B_B|B	2	Bimetallic
hollow|A_A_B|HCP	3	Bimetallic
hollow|A_A_B|FCC	3	Bimetallic
hollow|A_B_B|HCP	3	Bimetallic
hollow|A_B_B|FCC	3	Bimetallic
4fold|A_A_B_B	5	Bimetallic
top-tilt|A	1	Bimetallic
top-tilt|B	1	Bimetallic
bridge-tilt|A_A|B	2	Bimetallic
bridge-tilt|A_B|A	2	Bimetallic
bridge-tilt|A_B|B	2	Bimetallic
bridge-tilt|B_B|A	2	Bimetallic
bridge-tilt|B_B|B	2	Bimetallic
hollow-tilt|A_A_B|FCC	3	Bimetallic
hollow-tilt|A_A_B|HCP	3	Bimetallic
hollow-tilt|A_B_B|FCC	3	Bimetallic
hollow-tilt|A_B_B|HCP	3	Bimetallic

$$\Delta E_C = E_{slab+C} - E_{slab} - E_{CH_4} + 2E_{H_2} \tag{S2}$$

$$\Delta E_H = E_{slab+H} - E_{slab} - \frac{1}{2}E_{H_2} \tag{S3}$$

$$\Delta E_O = E_{slab+O} - E_{slab} - E_{H_2O} + E_{H_2} \tag{S4}$$

Where E_{slab+i} is the total energy of the surface with the adsorbate, E_{slab} is the energy total of the surface, and E_i is the total energy of the adsorbate. Common gas adsorbates were used as references for E_i because these reactions are more plausible than direct atomic adsorption.

S2.3. Feature Calculations from Property Data

The calculation of structural and electronic properties was previously discussion in Section S1. Elemental properties were collected from the references stated in Table S1. Since databases often only provide property information based only on atomic identity, these values were averaged in alloy surfaces. In the case of site elemental features, only the properties of the atoms in the adsorption ensemble were collected and averaged. The electron affinities, ionization energies, sublimation energies, molar volume, and lattice constant in alloys were approximated as the arithmetic means (Equation S5) of the property values of the atoms present in the adsorption ensemble.[43] On the other hand, the electronegativity and valence electrons in alloys were approximated as geometric means (Equation S6).[59] This is because although atomic properties in alloys are typically expressed as arithmetic means,[8,43] electronegativity and valence electrons follow the geometric mean law for electronegativity neutralization, postulated by Sanderson in 1951[63] and validated by Parr & Bartolotti in 1982[64].

$$P = \frac{\sum_i^{EAC} P_i}{EAC} \tag{S5}$$

$$P = \left(\prod_i^{EAC} P_i\right)^{\frac{1}{EAC}} \tag{S6}$$

Where P_i is the property of atom i in the adsorption site, EAC is the ensemble atom count, and P is the site property of the alloy. Meanwhile, surface atomic properties in alloys were also obtained as arithmetic means, but since only alloys of equal mixing were considered in this study, the alloy properties are simply the midpoints of the two atomic properties (Equation S7).

$$P = \frac{P_1 + P_2}{2} \tag{S7}$$

S3. Exploratory Data from Property Datasets

S3.1. General Trends from Theory

The relationships of features inside the dataset were explored and graphically represented to observe theoretical models and trends. First, periodical trends were observed by plotting features against the number of valence electrons present in the monometallic or bimetallic surface. Figures S2a-c illustrates how a decrease in valence electrons is correlated with a stronger (i.e., more negative), C-adsorption, H-adsorption, and O-adsorption, respectively. This trend is consistent with the principles behind the d-band model.

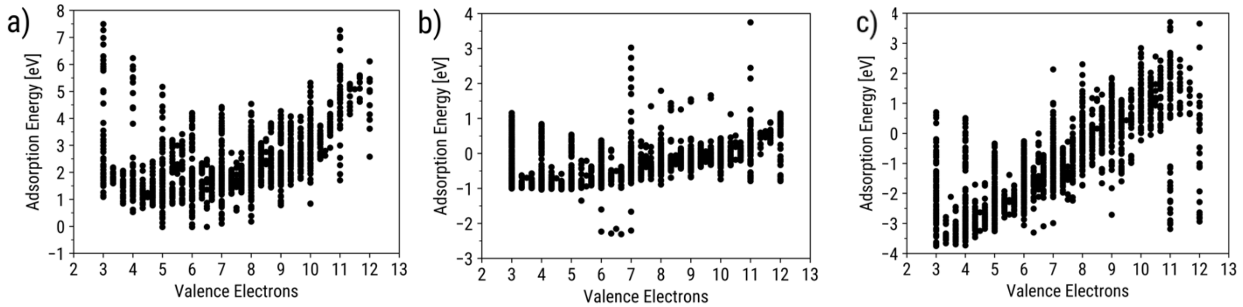

Figure S2. Relationship between valence electrons and a) C-adsorption energy, b) H-adsorption energy, and c) O-adsorption energy for TMs and TM alloys.

Figures S3a-c display illustrates how a increase in sublimation energy is correlated with a stronger (i.e., more negative), C-adsorption, H-adsorption, and O-adsorption, respectively. This trend is consistent with the principles behind the Friedel model, especially of C-adsorption.

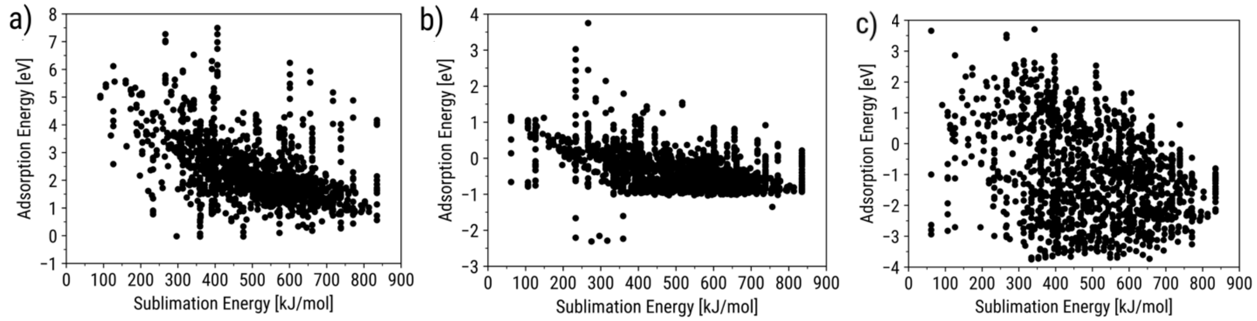

Figure S3. Relationship between sublimation energy and a) C-adsorption energy, b) H-adsorption energy, and c) O-adsorption energy for TMs and TM alloys.

*S3.**2**. Linear Scaling Relations from Common Descriptors*

The possibility of a direct linear relationship between common descriptors and adsorption energies was explored. Best fit lines were provided for the adsorption energies of monometallic transition metal surfaces, Cu-alloy surfaces, and Pt-alloys surfaces. This is because Cu is known as the standard transition metal for CO_2 conversion reactions[7] and Pt is the standard for fuel cell reactions.[4,6] Figures S4a-l illustrates these relationships between adsorption energy with respect to valence electrons, sublimation energy, d-band center, and d-band upper edge.

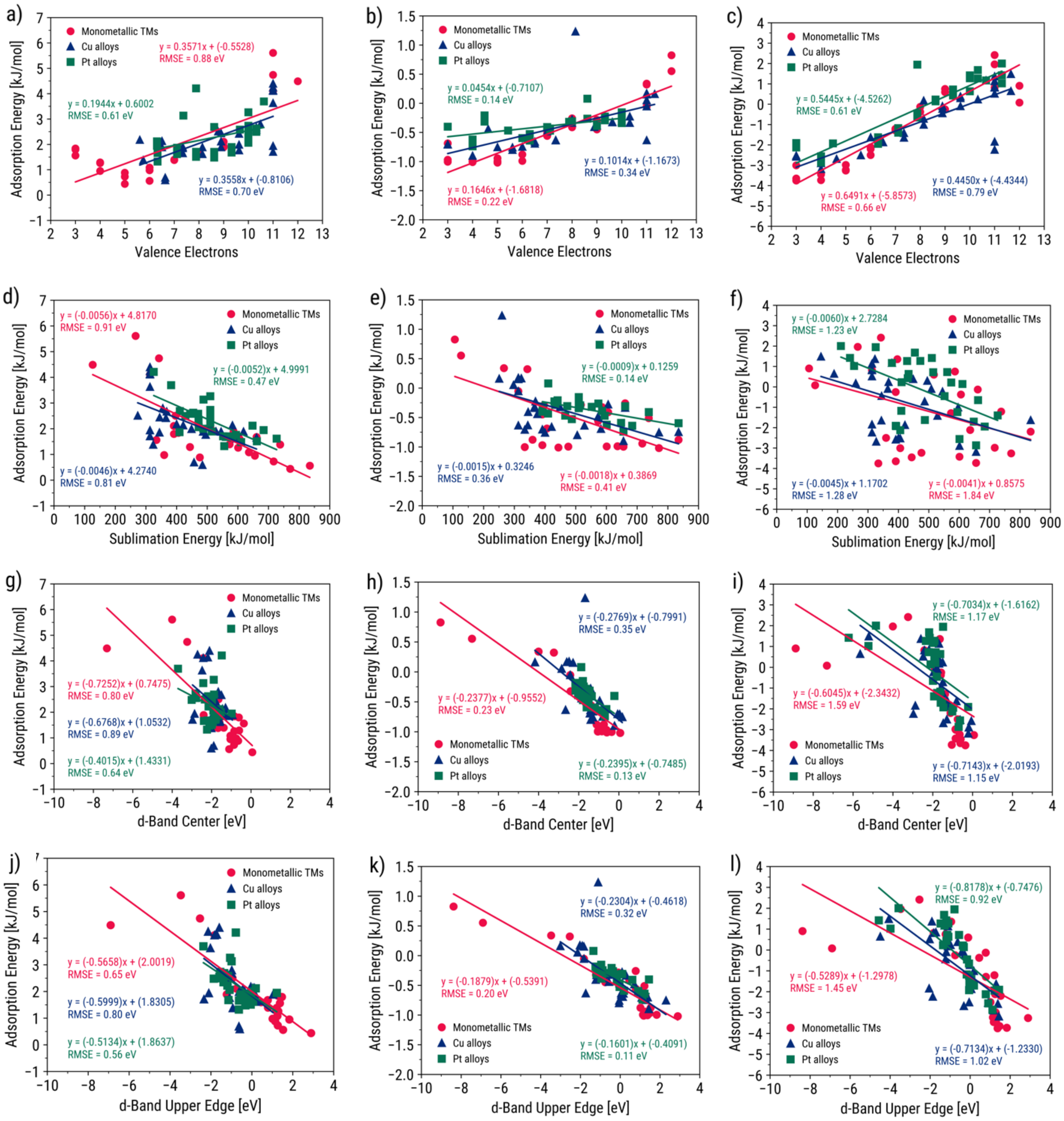

Figure S4. Linear scaling plots between adsorption energies and d-band model descriptors (**a-c**) valence electrons, (**d-f**) sublimation energy, (**g-i**) d-band center, and (**j-l**) d-band upper edge. C-adsorption energy prediction is in the first column, H-adsorption energy prediction in the second, and O-adsorption energy prediction in the third.

S4. Feature Engineering using Feature Selection

Table S3 details the results of Ward's Linkage and the representative feature of each multi-feature cluster.

Table S3. Retained and removes features from clusters identified by Ward's Method.

Adsorbate	Retained feature (correlation to adsorption energy)	Removed feature 1 (correlation to adsorption energy)	Removed feature 2 (correlation to adsorption energy)
*C	MV (-0.131)	WF (0.100)	-
	EA (0.207)	EN (0.137)	-
	DBC (-0.525)	VE (0.472)	IE (0.357)
	SE (-0.509)	DBW (-0.430)	-
*H	MV (-0.188)	WF (0.146)	-
	EN (0.225)	EA (0.183)	-
	DBC (-0.521)	VE (0.510)	IE (0.423)
	SE (-0.433)	DBW (-0.426)	-
*O	WF (0.576)	MV (-0.539)	-
	EN (0.609)	EA (0.450)	-
	VE (0.766)	IE (0.670)	DBC (-0.632)
	DBW (-0.512)	SE (-0.258)	-

The justifications behind these four clusters with highly correlated features are bound by physics-based concepts. First, Miedema et al. (1973) [39] discovered that there is a linear relationship between the square root of the molar volume and the electron density at the boundary of the Wigner-Seitz cell, which is the cell formed in reciprocal space during DFT calculations[17]. The work function is also a function of the Wigner-Seitz cell electron boundary thereby connecting the two features[40]. Second, electronegativity is correlated with electron affinity based on Parr & Pearson (1983)[41], who stated that electronegativity is the arithmetic mean between ionization energy and electron affinity. Third, the high correlation between the d-band center and the number of valence electrons is reflective of the d-band model[4]. The ionization energy is also dependent on the number of valence electrons, because higher valency results in a higher effective nuclear charge and therefore more ionization energy is needed to remove an electron.[42] Finally, sublimation energy and d-band width are highly correlated by virtue of the Friedel model, which states that the d-band width is used to calculate cohesive energy E_{coh} (Equation S9).

$$E_{\mathrm{coh}} = \left(\frac{q}{p} - 1\right)\left(\frac{DBW}{20}\right)(N_d)(N_d - 10) \tag{S9}$$

Where p and q respectively represent attraction and repulsion, while N_d is the number of d electrons in an atom. Since sublimation energy is highly correlated with cohesive energy due to their similar natures[65-66], sublimation energy has parabolic behavior as well.

S5. Atomic Adsorption Energy Prediction using ML Regression Models

S5.1. Regression Models and Hyperparameter Optimization

The three regression methods were chosen because these models each belong to a different category of regression models. Their strengths and weaknesses are expounded in Table S4. In the first stage of model training, the dataset was randomly split into 80% training data and 20% testing data. The hyperparameters of each model were optimized using Grid Search cross-validation, where all combinations of hyperparameters were evaluated to discover the best-performing model. The parameter grids indicating the combinations of hyperparameter values are displayed in Table S5. To prevent overfitting, the training set was further divided through a five-fold cross validation approach, as illustrated in Figure S5. The combination

of hyperparameter values with the lowest RMSE was chosen for further processing, as indicated in Table 2. These codes were ran under Python 3.11.8, with Keras 2.14.0, Scikit-learn 1.4.1.post1, Scikeras 0.12.0 and TensorFlow 2.14.1.

Table S4. Advantages and disadvantages of ML regression methods used in the study.

ML Model	RFR	GPR	ANN
ML algorithm category	Tree-ensemble	Kernel	Neural Networks
Working Principle	Hierarchical splitting of dataset for relationship mapping[67]	Non-parametric prediction from probabilistic model[29]	Construction of a multi-layer network of calculation nodes[67]
Advantages	Reduced overfitting[67]<br>Robust to outliers[67]	Compatible with small datasets[29]<br>Calculates uncertainty[29]	Highly customizable architecture[67]<br>Models non-linearities[67]
Disadvantages	Computationally expensive[67]<br>Black-box behavior[67]	Inefficient with high-dimensionality[29]	Computationally expensive[67]<br>Black-box behavior[67]

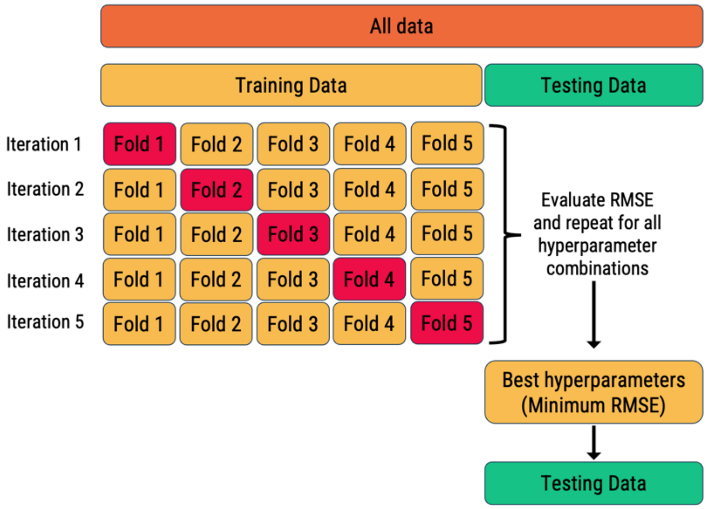

Figure S5. Schematic displaying the division of training, testing, and validation sets in the dataset using *k* folds. In this example, five-fold cross-validation was conducted (k = 5).

Table S5. Hyperparameter optimization of each regression model, and their respective grid-search ranges.

Model	Hyperparameter	Search Range
RFR	Number of trees in the random forest	[10, 50, 100, 150, 200]
	Maximum depth of tree	[None, 5, 10, 15, 20, 25]
	Number of features	$[N, sqrt(N), log_2(N)]$
GPR	Alpha	[10^{-10}, 10^{2}] (log-scale)
	Kernel	See list below
	Values for ConstantKernel( )	(10^{-2} to 10^{2})
	Length Scale for Matern( ), RationalQuadratic( ), and RadialBasisFunction( )	(10^{-2} to 10^{2})
	$Sigma_0$ for DotProduct( )	(10^{-2} to 10^{2})
	Noise level for White Kernel ( )	(10^{-8} to 10^{2})
ANN	Batch size	[5, 10, 15]
	Neurons in second hidden layer	[10, 15, 20]
	Neurons in third hidden layer	[0, 5, 10]
	Learning rate	[0.0001, 0.001, 0.01, 0.1]

The list of kernel functions is shown below:

- $\text{ConstantKernel( )} * \text{RBF( )} + \text{WhiteKernel( )}$
- $\text{ConstantKernel( )} * \text{RationalQuadratic( )} + \text{WhiteKernel( )}$
- $\text{ConstantKernel( )} * \text{Matern( )} + \text{WhiteKernel( )}$
- $\text{ConstantKernel( )} * \text{DotProduct( )} + \text{WhiteKernel( )}$
- $\text{ConstantKernel( )} * \text{RBF()} + \text{ConstantKernel()} * \text{RationalQuadratic()} + \text{WhiteKernel()}$
- $\text{ConstantKernel( )} * \text{RBF()} + \text{ConstantKernel()} * \text{Matern()} + \text{WhiteKernel()}$
- $\text{ConstantKernel()} * \text{RBF()} + \text{ConstantKernel()} * \text{DotProduct()} + \text{WhiteKernel()}$
- $\text{ConstantKernel()} * \text{RBF()} + \text{ConstantKernel()} * \text{RationalQuadratic()} + \text{WhiteKernel()}$
- $\text{ConstantKernel()} * \text{RationalQuadratic()} + \text{ConstantKernel()} * \text{Matern()} + \text{WhiteKernel()}$
- $\text{ConstantKernel()} * \text{RationalQuadratic()} + \text{ConstantKernel()} * \text{DotProduct()} + \text{WhiteKernel()}$
- $\text{ConstantKernel()} * \text{Matern()} + \text{ConstantKernel()} * \text{DotProduct()} + \text{WhiteKernel()}$

It should be noted that the inner hyperparameters of the kernels in GPR models (e.g., length scales, constant kernel sizes, white kernel sizes), were also optimized using the built-in limited memory Broyden-Fletcher-Goldfarb-Shanno algorithm for bounded ranges (L-BFGS-B) [68]. Another note for ANN models is that the first hidden layer of the model was set to 20 neurons, the number of epochs was set to 60, and the Rectified Linear Unit (ReLU) activation function was used. The contributions of the second and third hidden layers were to investigate non-linear effects.

S5.2. ML Model Accuracies and Benchmarking Details

The parity plots comparing the DFT-calculated adsorption energies and the ML-predicted adsorption energies of the finely-tuned models are shown in Figures S6a-i. Details of the different studies used for benchmarking are shown in Table S6.

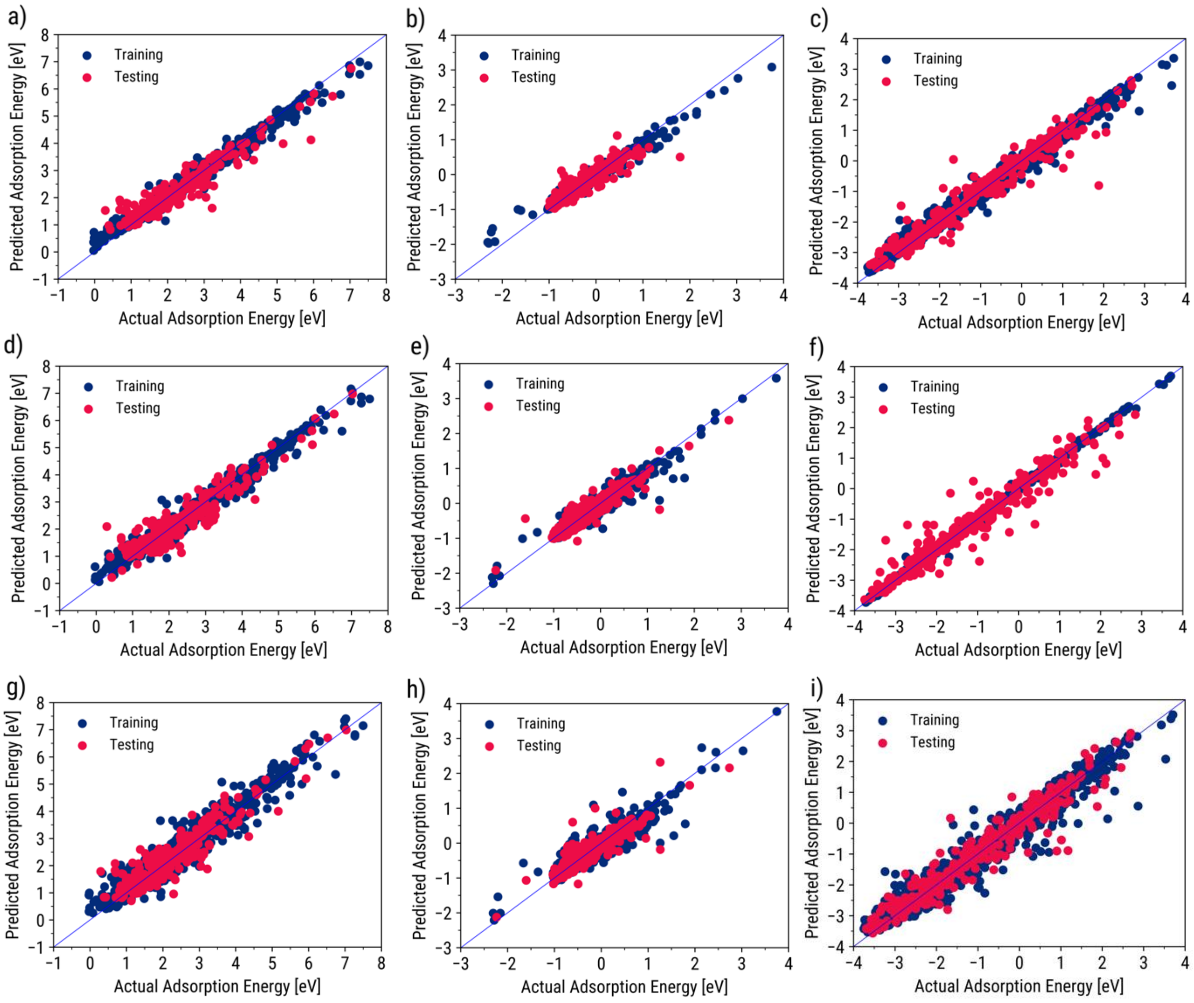

Figure S6. DFT-calculated vs ML-predicted parity plots of atomic adsorption energies using a-c) RFR, d-f) GPR, and g-i) ANN. a,d,g) *C, b,e,h) *H, and c,f,i) *O. C-adsorption energy prediction is in the first column, H-adsorption energy prediction in the second, and O-adsorption energy prediction in the third.

Table S6. Dataset information used in benchmarking ML-DFT approaches for adsorption energy prediction.

Study	Scope of Materials	Regression models	Data points considered in dataset
This work	Monometallic transition metals and alloys (1:1 mixing)	RFR, GPR, ANN	All adsorption sites
Tomacruz et al. (2022) [26]	Monometallic transition metals	RFR, GPR, ANN	All adsorption sites
T. R. Wang et al., (2020) [25]	Monometallic transition metals and alloys (1:3, 1:1, and 3:1 mixing)	RFR, ANN	Only the most stable adsorption sites (most negative adsorption energies)

S6. Feature Contributions from Interpretation Methods

Using the model-agnostic methods PFI and SHAP, global feature contributions and local feature effects were obtained from the trained models. Their working principles, strengths, and weaknesses are expounded in Table S7. It is important to use different interpretation methods because their definitions for feature contributions are different, and a consistent ranking of feature contributions can further support the claim for highest contributing features.

Table S7. Discussion of interpretation methods used.

	Permutation Feature Importance (PFI)	Shapley Additional exPlanations (SHAP)
Functions	Ranks features based on global importance	Ranks features based on global importance<br>Provides local feature effects
Definition of Feature Contribution	Accuracy drop (R^2) when a variable is corrupted[69]	Average marginal contributions of a variable to prediction (using game theory) [54]
Procedure	Comparison of R^2 values before and after shuffling the data of the corrupted variable[69]	Applies cooperative game theory to calculate marginal contributions across all possible subsets of features[54]
Model Agnostic?	Yes	Yes
Advantages	Computationally inexpensive<br><br>Less biased than forest interpretation methods towards continuous or categorical variables with high cardinality[69]	Graphically provides trends between feature values and predictions<br><br>Considers the interaction correlations between two features[54]
Disadvantages	Only provides global feature importances[69]<br>Effective only if input features are not highly-correlated[44]	Computationally expensive[53]<br>Ineffective for datasets with high dimensionality[44]

Global feature contributions were expressed by PFI as feature importances which were solved using Equation S10.

$$i_j = \mathrm{s} - \frac{1}{K}\sum_{k=1}^{K} s_{k,j} \tag{S10}$$

Where i_j is the individual feature score of variable j, s is the orginal R^2 score, $s_{k,j}$ is the new R^2 score after variable corruption, and K is the number of iterations.[54]

Unlike in PFI, which only evaluates the accuracy drop across the entire dataset with a corrupted feature j, marginal contributions in SHAP are evaluated across all possible subsets of features (coalitions). This ensures that interaction effects between two or more features are also accounted for, a quality not present in PFI.[54] First, the predictive model $f(x)$ is represented with an analogous explanation model g for the simplified input x', as seen in Equation S11.

$$f(x) = g(x') = \phi_o + \sum_{j=1}^{M} \phi_j x_j' \tag{S11}$$

Where ϕ_o is the null output (the average adsorption energy across the testing set), and ϕ_j is the Shapley value of feature j to represent feature effects. The simplified input x' is a binary vector of size M that represents the presence or absence of a feature. As a measurement of local feature effects, Shapley values provide directional insights on adsorption energy prediction given a high or low feature value.[54] The Shapley values of each sample i were calculated in Equation S12.

$$\phi_j^{(i)} = \sum_{z' \subseteq x' \setminus \{j\}} \frac{|z'|!\,(M - |z'|! - 1)!}{M!} \left[f_x^{(i)}(z' \cup \{j\}) - f_x^{(i)}(z') \right] \quad \text{(S12)}$$

Where $z' \subseteq x' \setminus \{j\}$ represents all z' coalition vectors where the non-zero entries are a subset of the non-zero entries in x' without the presence of feature j, $|z'|!$ is the number of present features in the coalition vector, M is the total number of features in the dataset, and $f_x^{(i)}$ provides the predicted adsorption energy values given the coalition vectors with $(z' \cup \{j\})$ or without (z') feature j. The local effects of each sample should display direct or indirect relationships between the features and adsorption energy, and these relationships were compared with theoretical trends to check for consistency.[54] In addition, global feature contributions were expressed by SHAP as average feature effects or the mean of the absolute Shapley values per feature (ϕ_j), as seen in Equation S13.

$$I_j = \frac{1}{n} \sum_{i=1}^{n} \left| \phi_j^{(i)} \right| \quad \text{(S13)}$$

The global feature importances for both PFI and SHAP were already shown in Figure 6 for finely-tuned GPR models, while global feature importances for finely-tuned RFR and ANN models are found in Figures S7 and S8, respectively. Finally, local feature effects for finely-tuned GPR models were already shown in Figure 7, while Figures S9 shows these for finely-tuned RFR and ANN models.

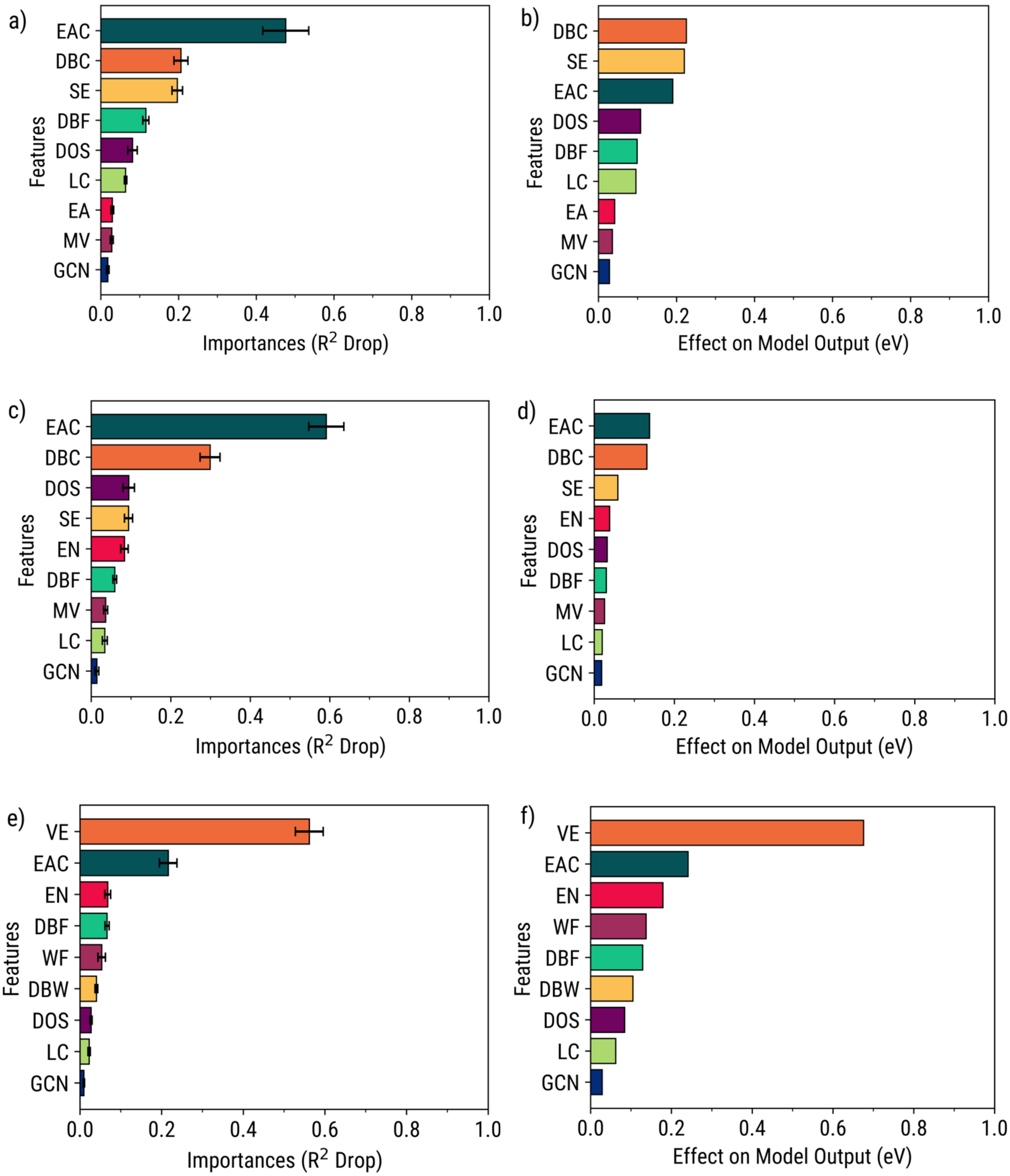

Figure S7. Feature importances in RFR models using interpretation methods: PFI [left] on C- (**a**), H- (**c**), and O- (**e**) property datasets, and SHAP [right] on C- (**b**), H- (**d**), and O- (**f**) property datasets.

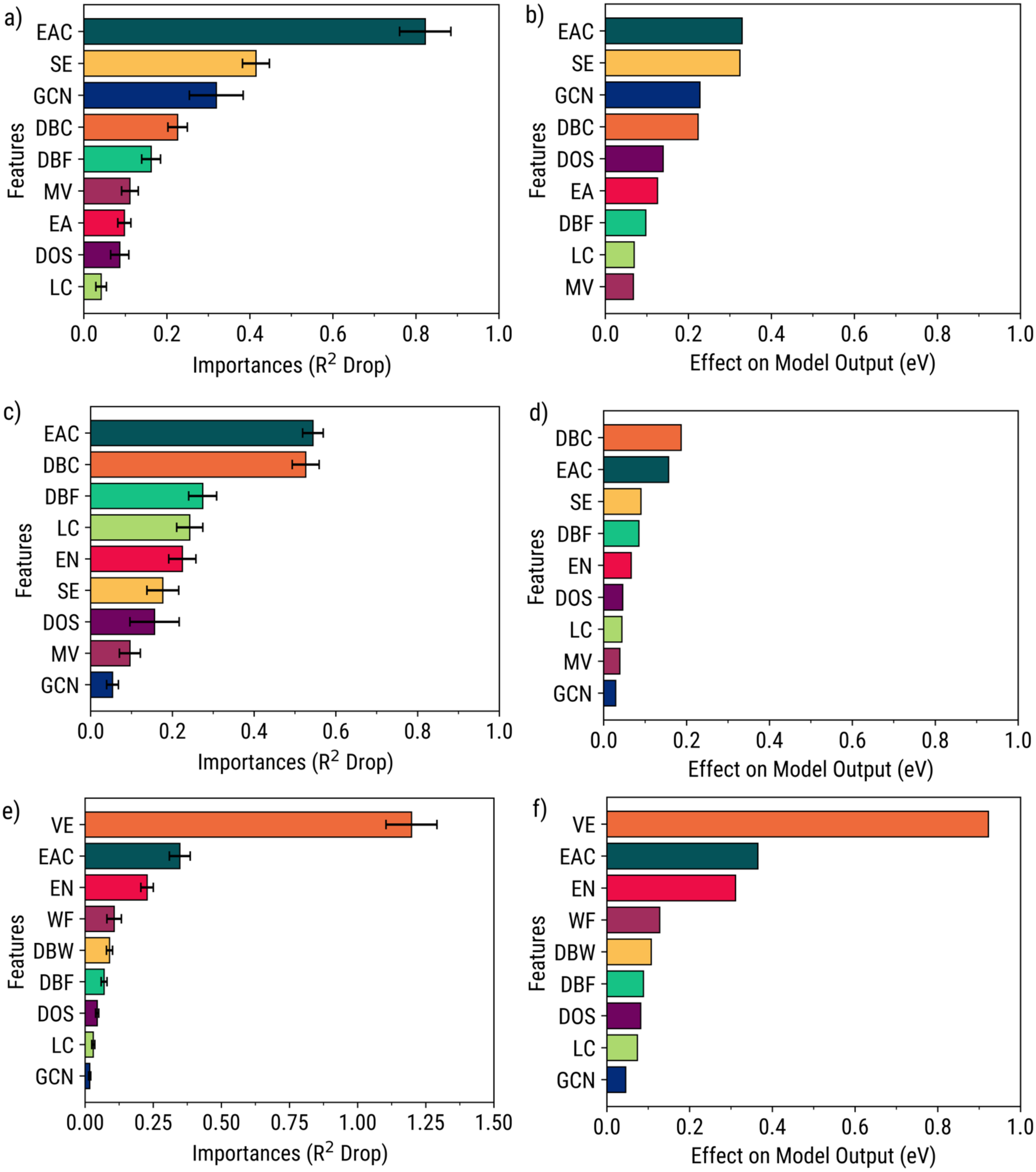

Figure S8. Feature importances in ANN models using interpretation methods: PFI [left] on C- (**a**), H- (**c**), and O- (**e**) property datasets, and SHAP [right] on C- (**b**), H- (**d**), and O- (**f**) property datasets.

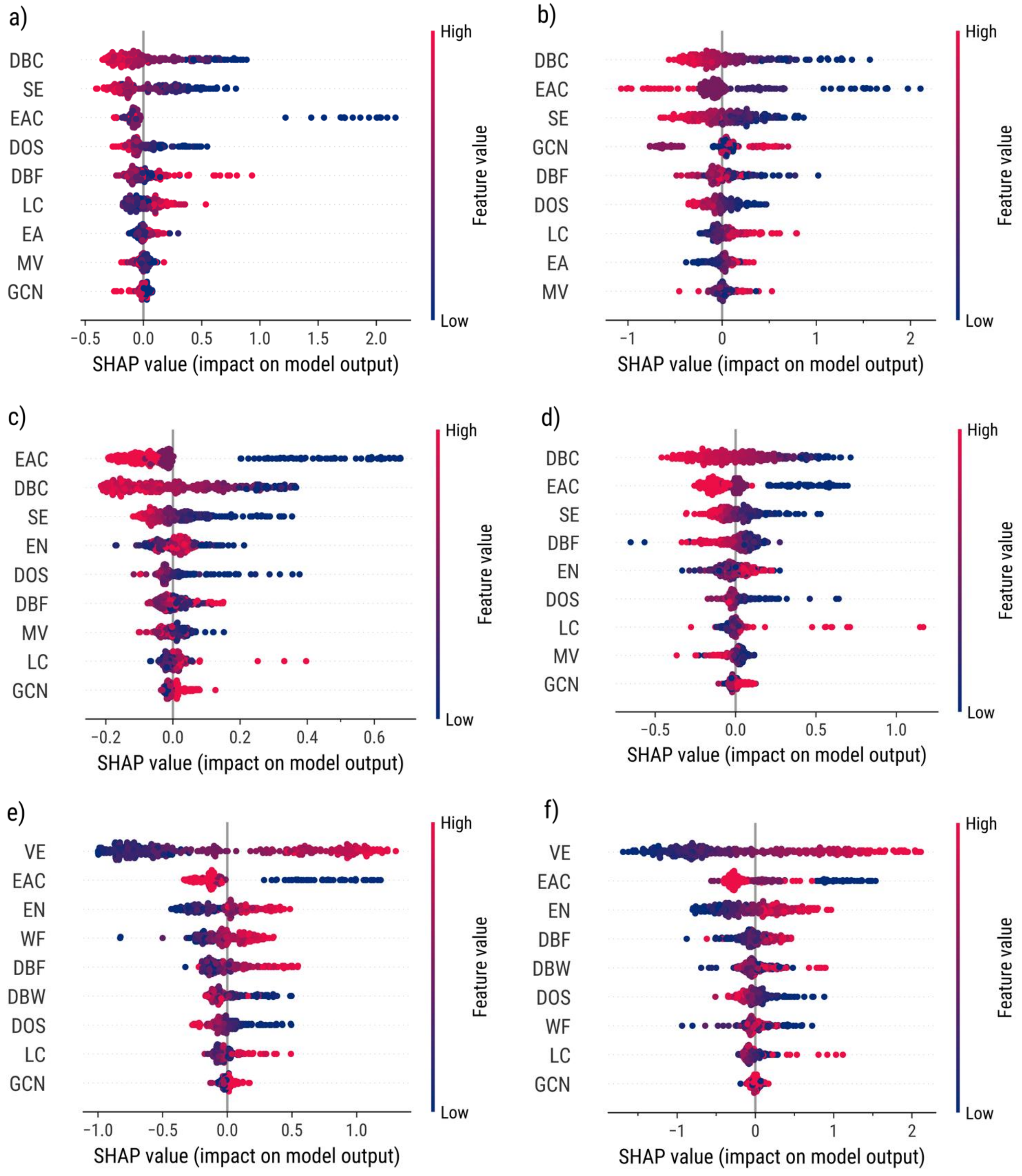

Figure S9. Local feature effect plots on (**a-b**) C-, (**c-d)** H-, and (**e-f**) O-adsorption energy predictions with RFR model [left] and ANN model [right] using SHAP.

References

[4] J. K. Nørskov, J. Rossmeisl, A. Logadottir, L. Lindqvist, J. R. Kitchin, T. Bligaard, H. Jónsson. *Journ. Of Phys. Chem. B.* **2004**, *108*(46), 17886-17892.

[6] J. K. Nørskov, T. Bligaard, A. Logadottir, J. R. Kitchin, J. G. Chen, S. Pandelov, U. Stimming. *Journ. Of The Electrochem. Soc.* **2005**, *152*(3), J23.

[7] X. Liu, J. Xiao, H. Peng, X. Hong, K. Chan, J. K. Nørskov. *Nat. Comms*. **2017**, *8*, 15438.

[8] J. K. Nørskov, F. Studt, F. Abild-Pedersen, T. Bligaard. *Fundamental Concepts in Heterogeneous Catalysis,* John Wiley & Sons, Inc., New Jersey, USA, **2014**, 87-137.

[15] O. Mamun, K. T. Winther, J. R. Boes, T. Bligaard. *Scientific Data.* **2019**, *6*(1), 1-9.

[32] K. T. Winther, M. J. Hoffmann, J. R. Boes, O. Mamun, M. Bajdich, T. Bligaard. *Scientific Data, 6*(1), 1-10.

[36] Scipy v1.13.0 Manual, "scipy.spatial.distance.squareform". **2024**, can be found under https://docs.scipy.org/doc/scipy/reference/generated/scipy.spatial.distance.squareform.html#scipy.spatial.distance.squareform, (accessed April 9, 2024), uploaded 2024 by SciPy API.

[38] T. Bock, "What is a Dendrogram? How to use Dendrograms", can be found under https://www.displayr.com/what-is-dendrogram/, (accessed August 13, 2022)**.**

[39] A. R. Miedema, F. R. De Boer, P. F. De Chatel. *Journal of Physics F: Metal Physics*, **1973**, *3*(8), 1558-1576.

[40] A. O. Christensen, *Proceedings of the IEEE International Vacuum Microelectronics Conference*, **1995**, 272-276.

[41] R. G. Parr, R. G. Pearson, *Journ. of the American Chem. Soc.,* **1983**, *105*(26), 7512-7516.

[42] J. Burdge, J. Overby. *Atoms First,* Mcgraw-Hill, USA, **2016**.

[43] M. Andersen, S. V. Levchenko, M. Scheffler, K. Reuter. *Catalysis*. **2019**, *9*(4), 2752-2759.

[51] A. Vojvodic, J. K. Nørskov, F. Abild-Pedersen, *Topics in Catalysis*, **2014**, *57*(1-4), 25-32.

[53] C. Molnar. *Interpretable Machine Learning: A Guide for Making Black Box Models Explainable*. **2019**, 154

[54] S. M. Lundberg, S. I. Lee. *NIPS'17: Proceedings of the 31st International Conference on Neural Information Processing Systems*. **2017**, 4768-4775.

[55] Quantum ESPRESSO. "Self consistency" can be found under https://www.quantum-espresso.org/faq/faq-self-consistency/#6.13 (accessed: August 13, 2022).

[56] QuantumATK. "The Hartree Potential" can be found under https://docs.quantumatk.com/manual/technicalnotes/hartree_potential/hartree_potential.html (accessed August 13, 2022).

[57] F. Calle-Vallejo, F., J. I. Martínez, J. M. García-Lastra, P. Sautet, D. Loffreda. *Angewandte Chemie International Edition*, **2014**, *53*(32), 8316-8319.

[58] W. Gao, Y. Chen, B. Li, S. P. Liu, X. Liu, Q. Jiang. *Nature Communications*, **2020**, *11*(1), 1196.

[59] Z. Li., S. Wang, W. S. Chin, L. E. Achenie, H. Xin, *Journ. of Mat. Chem. A*, **2017**, *5*(46), 24131-24138.

[60] [dataset] *CRC Handbook of Chemistry and Physics: A Ready-Reference Book of Chemical and Physical Data*, *102nd ed.* (Eds.: J. R. Rumble, T. J. Bruno, M. J. Doa), CRC Press/Taylor & Francis Group, Florida, USA, **2021**

[61] [dataset] Kramida, A., Ralchenko, Yu., Reader, J. and NIST ASD Team, **2020**, *Ionization Energies Form*, Atomic Spectra Database, Version 5.10, DOI**: 10.18434/T4W30F**

[62] [dataset] M. Winter, "The periodic table of the elements by WebElements", can be found under https://www.webelements.com, **1993** (accessed: October 12, 2021)

[63] R. T. Sanderson, *Science*, **1951**, *114*(2973), 670-672.

[64] R. G. Parr, L. J. Bartolotti, *J. Am. Chem. Soc.*, **1982**, *104*, 3801-3803.

[65] C. Baladron, M. P. Iñiguez, J. A. Alonso, *Solid-state Communications*, **1984**, *50*(6), 549–552.

[66] M. A. Turchanin, P. G. Agraval, *Powder Metallurgy and Metal Ceramics*, **2008**, *47*(1-2), 26-39.

[67] O. Theobald. *Machine Learning for Absolute Begineers,* **2017**, 77-99.

[68] R. H. Byrd, P. Lu, J. Nocedal, C. Zhu, *SIAM Journal on Scientific Computing*, **1995**, *16*(5), 1190–1208.

[69] C. Strobl, A.-L. Boulesteix, A. Zeileis, *BMC Bioinformatics,* **2007**, *8*, 25.

[70] Scikit-Learn v1.1.2 Documentation, "4.2. Permutation Feature Importance" can be found under https://scikit-learn.org/stable/modules/permutation_importance.html, (accessed April 9, 2024), uploaded 2024 by Scikit-Learn Documentation.
